# Authentication and Delegation for Operating a Multi-Drone System

**DOI:** 10.3390/s19092066

**Published:** 2019-05-03

**Authors:** Mungyu Bae, Hwangnam Kim

**Affiliations:** School of Electrical Engineering, Korea University, Seoul 02841, Korea; nardyen@korea.ac.kr

**Keywords:** Internet of Things, key management, authentication and delegation, drones control

## Abstract

As the era of IoT comes, drones are in the spotlight as a mobile medium of Internet of Things (IoT) devices and services. However, drones appear to be vulnerable to physical capture attacks since they usually operate far from operators. If a drone is illegally captured, some important data will be exposed to the attacker. In this paper, we propose a saveless-based key management and delegation system for a multi-drone control system. The proposed system enables a multi-drone control system to highly resist physical capture by minimizing exposure of confidential data. In addition, when the drone leaves the formation for performing another mission or by a natural environment, the system can allow the drone to securely re-participate in the formation with the help of the ground control station (GCS) when it comes back. The analysis result shows that the proposed system can reduce storage space usage and require less computational overhead. From the result, we expect that the system can guarantee the resistance of physical capture and secure key management to the drones as well as many mobile IoT devices.

## 1. Introduction

Unmanned aerial vehicle (or drone) technology has begun to be used in the private sector [1,2] due to technological advances such as control, communication, and cameras, unlike the past that was only used for military purposes. Drones with a higher degree of freedom can be used to provide various kinds of services [3]. For example, drones are widely used for shooting diverse sports activities at the individual level. Amazon is conducting research on using drones for book delivery [4], and New Zealand’s Domino pizza had succeeded in delivering pizza using drones [5]. This mobility raises the importance of employing one or more drones in the Internet of Things (IoT) environment [6,7]. Drone can provide surveillance [8] in the places or angles that humans are unable to reach due to the drone’s unlimited mobility, and it also can provide connectivity by improvising a local area network infrastructure in real-time according to the overall situation. In addition, the multi-drone system enables us to diffuse commands into and/or collect data from geographically-disperse sensor devices, which are essential functions in an IoT system, and thus, it can extend the battery life of sensor devices and reduce the cost of infrastructure deployment by collecting the data while moving.

However, there are limitations to controlling a drone based on a remote controller (RC) because it requires interaction with the controller, and it can be worse when multiple drones are employed in providing services. It is infeasible for one pilot to control several drones at the same time through multiple RCs. Even if several pilots operate drones one by one, the burden of labor costs will increase as the number of drones required for service increases. To make drone control autonomous and reduce control overhead from pilots, ground control station (GCS)-based control draws much attention in drone research and community [9]. Using features such as waypoint flight or setpoint flight in the GCS, a drone can autonomously move to the target point without any RC operation. The GCS also makes it easy to implement a multi-drone system and its formation.

Due to its performance and purpose of the drone, the altitude at which actual drones fly is at a level that humans can see with the naked eye. As a result, drones are likely to be exposed to other people and, even more, can be physically captured by hostile persons. Attackers can then inject false data into the captured drone, or directly control the drone as if it is a normal drone. Physical capture is actually meaningful. Note that many researchers are conducting studies to catch drones by birds or make a forced landing using radio waves. Considering most drones communicate with each other based on an ad-hoc network [10] in a multi-drone system, using the same group key for all the drones may expose all their session keys to the same hacking risk when one of the drones is captured. To show how dangerous group key usurpation is in a multi-drone system, we designed a simple scenario. In this scenario, we first extracted a secret key from the packets between the GCS and the multi-drone system with a common hacking tool. After that, we made a fake GCS and tried to send a command *disarm* with the extracted group key, which forces the drone to crash. The whole process is shown in our video (https://youtu.be/tw95S1KBlls/). Following the video, what we can see is that it is important to have strong resistance to physical capture to prevent group key exposure. The key update to prevent this exposure can cause a temporary disconnection of all the drones, which may result in a catastrophe in the control of a multi-drone system. Moreover, asymmetric key encryption based on a public key requires many operations and subsequently much computation, so it is better to exclude it if possible.

Consequently, a security scheme for a multi-drone system should have strong resistance to such physical capture. Moreover, using a symmetric key-based encryption is better than an asymmetric key-based algorithm due to the constrained resource on the drone. In this paper, we propose a saveless-based key management and delegation system for a multi-drone system. The meaning of saveless property is that the drone saves a minimum of secret data in its storage. In the proposed system, all group session keys are not the same, only drones which are same-hop from the GCS have the same group key, which is different from other group key management systems. These session keys are generated by the hash chain function, so one of the drones can know the other’s session key, and they can communicate with each other. When a physical capture occurs, which leads to changing the network topology, the GCS generates new session key and sends it to all drones except the captured drone. However, it is possible for a drone to leave the flight formation according to flight schedules or external factors, such as wind or flat battery. In order to compensate for this, the proposed system provides a readmission process for breakaway drones. We then propose the delegation system for GCS, which can be used in many scenarios. Complexity analysis shows that our algorithm can reduce storage usage and communication traffic.

The major contributions of our paper are:

- We propose the saveless-based drone authentication system, which is resistant to physically capture. Following the system, all drones in the network basically have a different session key, so secret data leakage can be minimized even when one drone is captured;
- We introduce the saveless-based drone delegation system. With our delegation system, the GCS can temporarily give the control right to another GCS with simple authentication.

The rest of the paper is as follows: We shows the related work in Section 2. The proposed saveless-based group key management system is shown in Section 3. Section 4 presents our drone delegation system. Section 5 analyzes how our system is secure. Section 6 shows the results of our system in numerical evaluation and simulation study based on actual experimental data. Finally, we conclude the paper in Section 7.

## 2. Related Work

Issues related to group key management have already been studied extensively. The advantage of a group key is the ability to reduce the key size since all members in the same group share one group key to communicate with each other. For example, if all connections require different keys, the number of required keys is $\frac{n(n-1)}{2}$. However, using the group key, the required key is only one. This idea is supposed to be used for networks whose nodes have limited calculating power and storage, such as sensor networks. Recent studies on the group key are based on attribute-based encryption [11,12,13,14]. Following this idea, it is possible to send the data to users who have appropriate attributes without additional checking attributes, unlike other algorithms. However, this idea is too heavy to directly apply to sensor networks or drone networks.

Our system is symmetric key-based key management, and movable node-based key management, which seems like a network dependent-based scheme [15]. A network-dependent approach basically supports node moving over a widely distributed wireless network. The main point of this environment is re-keying [16], which is introduced as a key renewal process in the proposed system. For re-keying, there are some studies to reduce the re-keying overhead. Sandirigama et al. [17] used password-based key management with a combination of re-keying and authentication. Suganthi et al. [18] proposed the energy efficient key management scheme by generating pair-wise keys and maintaining them without key sharing. However, these algorithms are not suitable for the drone environment.

The proliferation of the IoT network, which includes devices which have small size and limited performance but can be highly distributed with an ad-hoc-based wireless network, has been highlighted. These devices are basically operated on a group basis; the network operating environment is similar to the drone environment. Li et al. [19] showed the secure sensor association and key management scheme in a body area network. A group of nodes establish group pairing before the deployment, then the secret keys are generated. However, the difference with the body area network is that each node in the drone network moves to a different path. Zhang et al. [20] were able to filter the false data through group rekeying which is sent by the compromised node. Following this idea, the future key cannot be extracted without neighbor nodes, but it is not sufficient to satisfy mobility, which is important for the drone environment.

The self-healing key distribution scheme [21,22,23] is useful when network status is unstable in the wireless network. In a drone environment, the self-healing property seems important since all connections are based on an ad-hoc wireless network and all nodes continuously move in 3D-based directions. However, the property is vulnerable to physical capture. Following this property, the node stores some information to restore the session key. When the node is physically captured, all data in the storage will be disclosed. In the proposed system, the only exposed data are the current session key, and the topology and session key will be changed when the system notices the physical capture attack.

## 3. Saveless-Based Group Key Management System

In this section, we propose a saveless-based group key management system to guarantee resistance to physical capture. This system consists of four protocols: Initial key management, node communication, group key renewal, and withdrawal management. These protocols are described in detail below.

Before introducing the protocols, some assumptions and the environments of multi-drone flight control are as follows: All drones and GCSs are connected with Wi-Fi-based ad-hoc networks. The GCS is the administrator which gives various commands and sets the course of the drones. The *n* drones fly following the flight path and execute the command by the GCS. The drones are located in the proximity of the GCS before the flight, which means that initial group key generation and distribution protocols are secured.

### 3.1. Initial Group Key Generation and Distribution Protocol

This protocol will be run before the initiation of the flight, and all communication is secured. When a key generation protocol is finished, the GCS sends the group key $k_{0}^{1}$ to drones selected by prearranged topology with one-hop distance, since the GCS and all drones are not in operation. The drones, which receive their group key $k_{0}^{1}$, calculate following each of their next-hop node’s group key and then send the key to next-hop drones.

(1) $$k_{i}^{h+1}=h\left(k_{i}^{h}\right),$$

where *i* will be 0 in this protocol. The idea is that the group symmetric key belongs to each drone and is different compared to other drones except the same-hop drone.

### 3.2. saveless-Based Communication Protocol

After the registration is over, the GCS can send some command messages, such as take-off and move to the setpoint, to the drone formation. The command message sent to the drone has to be encrypted with the group key. There are two possible types of communication protocol: GCS-to-drone broadcasting or node-to-node direct communication. Table 1 shows notations for this protocol and the group key renewal protocol.

#### 3.2.1. GCS-to-Drone Broadcasting Protocol

In the case of broadcasting, this type of communication is commonly used when the GCS sends the command message to the drone formation. There are two possible methods to broadcast the message: single node-based broadcasting, and forwarding-based broadcasting. Single node-based broadcasting is that a sender encrypts messages using each receiver’s group key. After that, the sender sends the appropriate message to the receiver one by one. This method seems common at our scheme, but it requires lots of overhead in the sending phase.

The forwarding-based broadcasting method is that a sender only forwards the encrypted message to next-hop receivers. The whole procedure is as follows.

1. A sender encrypts the message using next-hop’s group key in session *i*:
  (2) $$emsg=E_{s}\left(msg,k_{i}^{h+1}\right);$$
2. The sender forwards emsg to next-hop receivers;
3. The next-hop receivers get the msg decrypting esmg with their key $k_{i}^{h+1}$:
  $$msg=D_{s}\left(emsg,k_{i}^{h+1}\right);$$
4. The next-hop receivers reiterate the previous process, such as re-encrypt msg, using the next-hops’ session key and forward the result to their next-hop nodes.

As a result, all nodes in the network can receive the sender’s message. The encrypt function can be a commonly used symmetric encryption algorithm, such as DES or AES. The method can reduce the sending overhead compared to the previous method, and the energy consumption of intermediate drones can be also reduced since intermediate drones send the data at one time and symmetric key-based encryption and hashing does not need many computation power.

#### 3.2.2. Node-to-Node Direct Communication Protocol

The overall process of direct communication is almost the same as the broadcasting type. A different point is that the hop distance between a sender and a receiver may be higher than one. In this case, the temporal encryption key between two nodes will become the group key value of the node, which has higher hop distance. The reason is that the node which has a lower hop number can easily calculate the higher one’s group key using the hash chain. Following Equation (2), the message sent to the receiver is as follows:

(3) $$emsg=E_{s}\left(msg,h^{\left|sh-rh\right|}\left(k_{i}^{\min\left(sh,rh\right)}\right)\right),$$

where sh is the sender’s hop number, and rh is the receiver’s hop number. When two drones which are physically connected want to communicate with each other before the key renewal protocol activation, it is possible to share their hop count in this session.

### 3.3. Group Key Renewal Protocol

All drones in the network send a beacon message to announce their own network status. From the beacon message, the drone can see the need to update the network topology due to the position change of some drones. In this case, all group keys of the drones should be renewed to satisfy the saveless property. The member drone sends a message that requests a routing table and group key renewal to the GCS. Then, the GCS calculates a new session’s group key as follows:

(4) $$k_{i+1}^{0}=h\left(k_{i}^{0}\left\|r\right\|i+1\right).$$

To send the new session’s group key, the GCS determines which drone is the nearest one using the general routing algorithm and reported drones’ position. The GCS then wants to forward the new group key to the new 1-hop drone; there are the following issues in broadcasting the renewed key.

- The new group key has to be encrypted to send them because all drones are not close-range, in contrast to the initial key distribution protocol. Therefore, the GCS cannot use near communication or secured channel communication in this case;
- The key which will be used to encrypt should be an old group key since all drones do not have the new session’s group key;
- The position of all drones and routing paths is already changed, so the next-hop node may not have the $k_{i}^{h+1}$ when the drone’s old group key is $k_{i}^{h}$.

**Figure 1:**
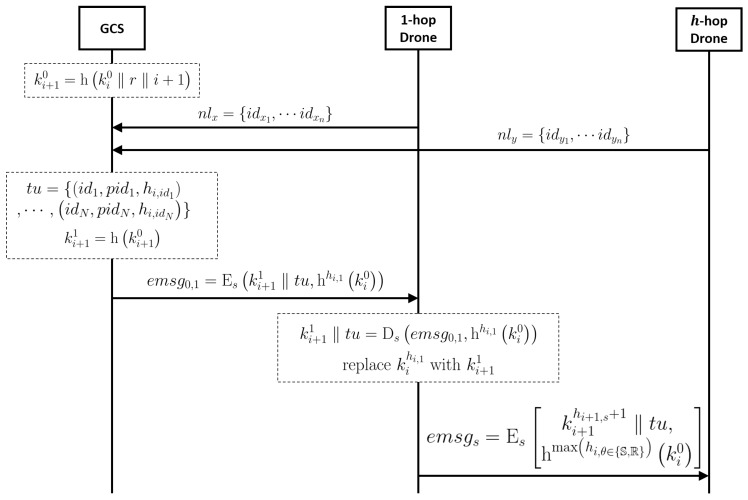
The procedure of the group key renewal protocol.

The whole procedure of renewed group key distribution considering previous issues is as follows (see also Figure 1).

**Figure 2:**
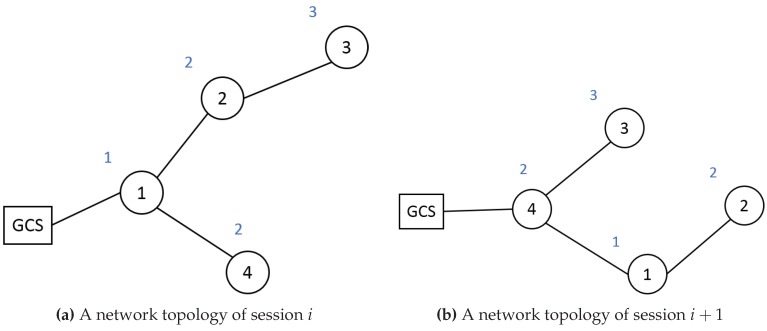
Example of the key renewal protocol.

1. Each drone periodically senses all neighbor drones;
2. When a drone notices that the neighbor drones has been changed, it broadcasts a request message that all drones need to report the list of their neighbor drones to the GCS;
3. The GCS collects the neighbor list of each drone, then calculates the new routing paths;
4. The GCS generates the following tuple tu:
  $$\begin{array}{c} tu=\{\left(id_{1},pid_{1},h_{i,id_{1}}\right),\cdots ,\left(id_{N},pid_{N},h_{i,id_{N}}\right)\}. \end{array}$$
  Figure 2 shows an example of the tuple. In the figure, a circle represents a drone *x*, a number in the circle is $id_{x}$, and a blue colored text above the circle is $h_{i,id_{x}}$. When network topology has been changed like in Figure 2b, the tuple tu generated by the GCS is $tu=\left\{\left(1,4,1\right),\left(2,1,2\right),\left(3,4,3\right),\left(4,0,2\right)\right\}$;
5. The GCS calculates its new session key $k_{i+1}^{0}$ following Equation (4) and next-hop’s new session key $k_{i+1}^{1}=h\left(k_{i+1}^{0}\right)$;
6. The GCS encrypts the $k_{i+1}^{1}$ and tu by Equation (3) to forward the result message to the next-hop node. In the example, the encrypted message $emsg_{0,4}$ which will be sent to drone 4 by the GCS is $emsg_{0,4}=E_{s}\left(k_{i+1}^{1}\|tu,h^{2}\left(k_{i}^{0}\right)\right)$, because drone 4’s old hop count is 2;
7. The nodes decrypt the encrypted message to get the new session key $k_{i+1}^{1}$ and find their next-hop nodes. In the example, drone 4 decrypts $emsg_{0,4}$ and gets $key_{i+1}^{1}$ using its old session key $k_{i}^{2}$. After that, drone 4 can find that the next-hop nodes are drone 3 and drone 1 by checking $pid_{x}$ in tu;
8. The nodes encrypt the new session key for their next-hop nodes as the following equation:
  (5) $${emsg}_{s}=E_{s}\left(k_{i+1}^{h_{i+1,s}+1}\|tu,h^{\max\left(h_{i,\theta \in \left\{S,R\right\}}\right)}\left(k_{i}^{0}\right)\right),\;\forall s\in S,\forall r\in R.$$
  All the next-hop nodes of the GCS just broadcast the message $\left\{h^{\max\left(h_{i,\theta \in \left\{S,R\right\}}\right)},emsg_{s,r}\right\}$ to their next-hop nodes. The next-hop nodes can decrypt $emsg_{s}$ since they know the id of the sender and session *i*’s hop count for all nodes. In the example, drone 4 has to broadcast a new session key to drone 3 and drone 1. Therefore, the encrypted message which drone 3 and drone 1 will receive is as follows:
  $${emsg}_{4}=E_{s}\left(k_{i+1}^{2}\|tu,h^{\max\left(2,1,3\right)}\left(k_{i}^{0}\right)\right);$$
9. All drones in the drone flight formation repeat the above procedures from step (7) to step (8), then all drones can get the new group key in session i+1.

### 3.4. Node Withdrawal and Re-Participation Protocol

There are two cases where a new node participates in the drone flight formation already in operation: When another GCS delegates its drone’s control, or when a drone is readmitted into the formation of the intended flight. Since a delegation case is explained in a later section; the readmission case is introduced in this subsection.

In the following flight environment or pre-populated scenario, some drones can break out of the formation. The factors that can affect the flight route of the drone are not only a natural environment, but also built environment, such as winds, rain, the position of the building or trees, etc. Some drones may be disconnected from the flight formation because of these situations. The breakaway drones can lead back to the right position of the formation due to the adjustment of an attitude control system.

The breakaway drones attempt to re-join the drone network but will be denied. First, the drones have to prove that they were not physically captured while they departed from the network. Even though this proof has been accepted, the drones cannot accept the new group key from the proposed group key renewal protocol since they do not have the group key of the last session. The GCS may change the session and run the group key renewal protocol at the moment of confirmation that some drones left the network. Initializing the session and distributing the initial group key is too dangerous in action.

Even though there are some solutions to re-authenticate the drone with a symmetric-based encryption scheme, a new re-authentication protocol is needed since it is hard to distinguish the cases between the physical capture or scheduled/adventitious breakaway. This is because our protocol guarantees saveless property to defend the physical capture. The scheme for re-authentication consists of two parts, the withdrawal and the readmission part. Table 2 shows notations used in the withdrawal and re-participation scheme. The whole procedures of the withdrawal part are as follows.

1. When the breakaway drone $N_{b}$ is aware that the link between itself and the other drones in a formation is broken, it rapidly runs the encryption algorithm to save the information, where *r* is a random nonce:
  $$ed_{1}=E_{s}\left(id_{b}\|fd_{1}\left\|t_{w}\right\|r_{b},k_{i}^{h}\right),r_{b}=h\left(t_{w}\|r\right);$$
2. Further, it immediately replaces the key $k_{i}^{h}$ with temporary key ${\hat{k}}_{1}^{h}=h\left(k_{i}^{h}\|fd_{1}\right)$, and the flight data $fd_{1}$ with $ed_{1}$;
3. After some period of time, $N_{b}$ repeatedly generates encrypted flight data $ed_{j+1}$ and a new temporary key ${\hat{k}}_{j+1}^{h}$ following equations, where *j* means a temporary session number:
  $$ed_{j+1}=E_{s}\left(fd_{j+1},{\hat{k}}_{j}^{h}\right),\;{\hat{k}}_{j+1}^{h}=h\left({\hat{k}}_{j}^{h}\|r_{b}\right);$$
4. $N_{b}$ replaces the old temporary key with a new temporary key at the indicated intervals but saves all encrypted flight data $ed_{1},\cdots ,ed_{\lambda }$, and timestamp $t_{w}$ when $N_{b}$ is far away.

**Figure 3:**
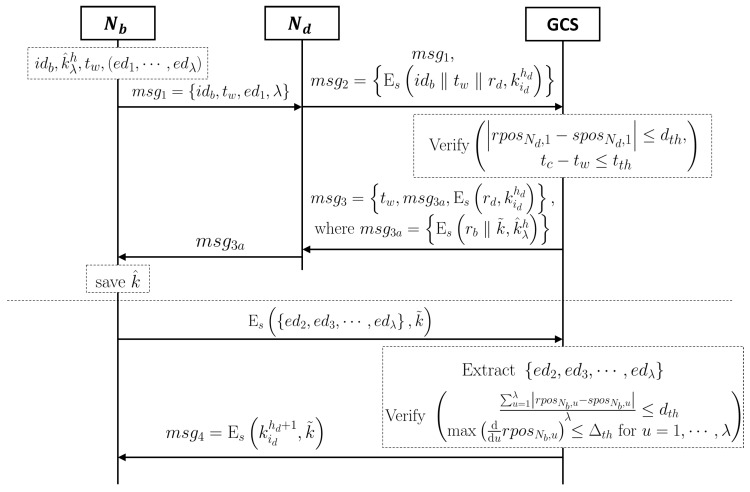
The procedure of the node’s withdrawal and re-participation protocol.

The readmission part consists of two processes: The pre-verification process and the readmission process. Figure 3 shows the whole readmission part. When $N_{b}$ re-meets its original formation, a pre-verification process will be started as follows.

1. $N_{b}$ sends the verification request message $msg_{1}=\left\{id_{b},t_{w},ed_{1},\lambda \right\}$ to a first node $N_{d}$ which discovers $N_{b}$;
2. $N_{d}$ sends $msg_{1}$ and $msg_{2}=\left\{E_{s}\left(id_{b}\left\|t_{w}\right\|r_{d},k_{i_{d}}^{h_{d}}\right)\right\}$ to the GCS, where $r_{d}$ is a random nonce of $N_{d}$, $h_{d}$ is a hop number of $N_{d}$, and $i_{d}$ is a current session number of $N_{d}$;
3. The GCS extracts the $ed_{1}$ from $msg_{1}$ since it knows the session when $N_{b}$ has been left the formation;
4. The GCS verifies the real position of $N_{d}$ at $t_{w}$:
  $$\left|rpos_{N_{d},1}-spos_{N_{d},1}\right|\leq d_{th};$$
5. Further, the GCS verifies the timestamp $t_{w}$ by $t_{c}-t_{w}\leq t_{th}$, where $t_{c}$ is a current time;
  - If the withdrawal is scheduled, the GCS can estimate the $t_{c}-t_{w}$, then $t_{th}$ will be set to an appropriate time;
  - If the withdrawal reason is uncertainty, such as wind and obstacles, $t_{th}$ has to be very short.
  If the two verification processes are completed, the GCS orders $N_{d}$ and $N_{b}$ to run a one-time key exchange protocol.
6. The GCS sends a message $msg_{3}$ to $N_{d}$ as follows:
  $$\begin{array}{c} msg_{3}=\left\{t_{w},msg_{3a},E_{s}\left(r_{d},k_{i_{d}}^{h_{d}}\right)\right\},\\ \mathrm{where}\;msg_{3a}=\left\{E_{s}\left(r_{b}\|\tilde{k},{\hat{k}}_{\lambda }^{h}\right)\right\}; \end{array}$$
7. $N_{d}$ saves $\tilde{k}$, then sends the $msg_{3a}$ to $N_{b}$;
8. $N_{b}$ decrypts the received message and saves $\tilde{k}$.

In this process, the GCS can know the ${\hat{k}}_{\lambda }^{h}$, because following equation ${\hat{k}}_{\lambda }^{h}=h^{\lambda -1}\left(h\left(k_{i}^{h}\|fd_{1}\right),r_{b}\right)$, the GCS already knows $k_{i}^{h}$ and receives $fd_{1}$ and $r_{b}$ from $ed_{1}$. $N_{b}$ cannot communicate with the GCS since it does not have the old session key $k_{i}^{h}$. The above algorithm gives the one-time session key used to complete a readmission process. The readmission process involves the following steps:

1. $N_{b}$ sends $cmsg=E_{s}\left(\left\{ed_{2},ed_{3},\cdots ,ed_{\lambda }\right\},\tilde{k}\right)$;
2. The GCS extracts $\left\{ed_{2},ed_{3},\cdots ,ed_{\lambda }\right\}$ from cmsg;
3. The GCS verifies following two conditions:
  $$\frac{\sum_{u=1}^{\lambda }\left|rpos_{N_{b},u}-spos_{N_{b},u}\right|}{\lambda }\leq d_{th}$$
  $$\max\left(\frac{d}{du}rpos_{N_{b},u}\right)\leq \Delta_{th}\;\mathrm{for}\;u=1,\cdots ,\lambda ,$$
  where the first condition means how the breakaway drone has followed the correct route on average, and the second condition means whether or not the breakaway drone sharply changes its position by virtue of the attacker’s physical capture;
4. If the two conditions are successfully passed, the GCS sends a message which encrypts its new session key, $\left\{E_{s}\left(k_{i_{d}}^{h_{d}+1},\tilde{k}\right)\right\}$, to $N_{b}$;
5. $N_{b}$ revokes $\tilde{k}$.

## 4. Drone Delegation System

**Figure 4:**
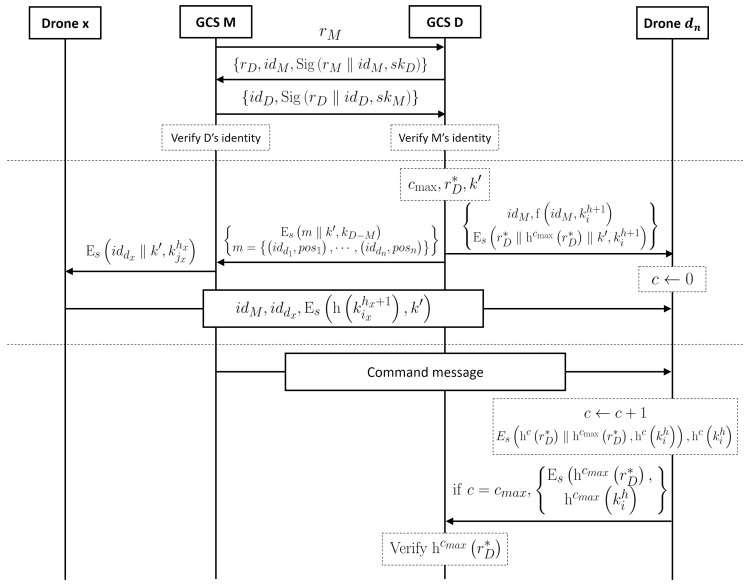
The procedure of the drone delegation system.

According to the operational scenarios, a drone flight formation can be controlled by multiple GCSs. Moreover, some GCSs can temporarily or permanently delegate the control of the drone flight when the range of operations is very large. In this case, drone delegation and ownership transfer are important things in both drone security and security performance. The drone delegation system requires two protocols: The GCS authentication protocol and the drone delegation protocol. Table 3 shows the notation used in this section. Figure 4 shows the whole procedure of the drone delegation system.

### 4.1. GCS Authentication Protocol

The whole procedure in which a delegator GCS and a mandatory GCS authenticate each other is as follows:

1. The delegator GCS *D*, which delegates its drones to the mandatory GCS *M*, first requests delegation to *M* sending $id_{D}$;
2. *M* sends random nonce $r_{M}$ to *D*;
3. *D* sends a message $\left\{r_{D},id_{M},\mathrm{Sig}\left(r_{M}\|id_{M},sk_{D}\right)\right\}$ to *M*;
4. *M* sends a message $\left\{id_{D},\mathrm{Sig}\left(r_{D}\|id_{D},sk_{M}\right)\right\}$ to *D*;
5. Each of *M* and *D* uses the other side’s public key, $pk_{D}$ and $pk_{M}$, to verify the other side’s identity, where the public keys can be gotten from the key master which manages the GCS’s public key.

### 4.2. Delegation Protocol

After the authentication of GCS, the next phase is drone delegation, and the mandatory GCS can control the drones with its key. An important point of the delegation is that this protocol temporarily controls the right to the mandatory GCS.

There are two cases in this protocol; the drone group belonging to *D* is far away from the group of *M* and the close case. The far case is very simple because *M* sends a command message to the *D*, in which the message is encrypted with shared key between *D* and *M*. The close case, however, *M* can directly send a command message to drones belonging to *D*. A procedure of the close case is as follows:

1. *D* decides $c_{\max}$, $r_{D}^{*}$, and replacement key $k^{\prime }$;
2. *D* broadcasts the following message to all of the drones under its command, where *i* is the current session of *D*:
  $$\left\{id_{M},E_{s}\left(r_{D}^{*}\left\|h^{c_{\max}}\left(r_{D}^{*}\right)\right\|k^{\prime },k_{i}^{1}\right),f\left(id_{M},k_{i}^{1}\right)\right\};$$
3. Some drones, which receive the broadcast message, check if $id_{M}$ is true by a keyed hash function, and save the message, then generate a forwarding message and forward it to their next-hop drone, where $\alpha =\left\{r_{D}^{*}\left\|h^{c_{\max}}\left(r_{D}^{*}\right)\right\|k^{\prime }\right\}$:
  $$\left\{id_{M},E_{s}\left(\alpha ,k_{i}^{h+1}\right),f\left(id_{M},k_{i}^{h+1}\right)\right\};$$
4. Repeat (2) until all drones receive the message;
5. *D* sends the following message to *M*, where $id_{d}$ is the drone’s id, and *n* is the number of drone under *D*:
  $$E_{s}\left(\left\{\left(id_{d_{1}},pos_{1}\right),\cdots ,\left(id_{d_{n}},pos_{n}\right)\right\}\|k^{\prime },k_{D-M}\right);$$
6. *M* calculates the new route for the delegated drones;
7. *M* sends the following key renewal message to all nodes in A, where $id_{d_{x}}$ is a delegated pair of drone *x*:
  $$\left\{E_{s}\left(id_{d_{x}}\|k^{\prime },k_{j_{x}}^{h_{x}}\right)\right\},\forall x\in A;$$
8. Each drone in A decrypts the received message, then it sends the following message to their pair delegated drone for distributing a new session key:
  $$\left\{id_{M},id_{d_{x}},E_{s}\left(h\left(k_{i_{x}}^{h_{x}+1}\right),k^{\prime }\right)\right\},\forall x\in A;$$
9. The delegated nodes check $id_{M}$ using the keyed hash function and save key $k^{\prime }$;
10. If the previous hop node of a delegated node is also a delegated node, then the previous node runs step (8) to act as a node in A.

### 4.3. Delegation-Based Command Protocol

Since *M* receives the command right temporarily in our delegation system, delegated drones have to send the update message to the *M*. Like many delegation protocols, we adopted the parameter as a command counter, rather than a time duration. In drone control, there is a risk of loss of control in some cases if control is provided based on duration. The process of sending and receiving commands is as follows:

1. When the delegated node receives a command message, it replaces the stored encrypted message following equation:
  $$\left\{E_{s}\left(h^{c}\left(r_{D}^{*}\right)\|h^{c_{\max}}\left(r_{D}^{*}\right),h^{c}\left(k_{i}^{h}\right)\right),h^{c}\left(k_{i}^{h}\right)\right\};$$
2. If $c=c_{\max}$, then the delegated node notes to *M* that delegation has to be renewed with $\left\{E_{s}\left(h^{c_{\max}}\left(r_{D}^{*}\right)\right),h^{c_{\max}}\left(k_{i}^{h}\right)\right\};$
3. *M* sends the received message to *D*;
4. *D* verifies if delegation renewal is required by checking $h^{c_{\max}}\left(r_{D}^{*}\right)$ since *D* has $r_{D}^{*}$ and $k_{i}^{h}$;
5. If this verification succeeds, then *D* and *M* run the delegation protocol again.

## 5. Security and Usability Analysis

In this section, we show that the proposed algorithm is secure against possible attacks in a drone environment. The above four items in the table show the security analysis; the other four items represent how these systems are suitable for a drone environment.

### 5.1. Physical Capture Attack

As we have already noticed in the introduction and related work, the drone is easy for the attacker to capture. Following our protocol, exposed data in the drone are as follows when the drone is captured: Hashed session key, encrypted flying data of the drone, and the timestamp when the node has been hijacked. When the protocol uses the cryptographic hash function, the attacker cannot know the last session key due to the one-way property of the hash function. The encrypted flying data are protected with the last session key, as the attacker who does not know the last session key cannot decrypt this message. Even though the attacker can extract the session key from the captured drone, the network topology and session key will be updated when the capture attack is detected. After that, the extracted session key is of no use. Overall, the riskiness of key exposure depends on the security of cryptographic hash functions.

The easy method for attacking the proper network after the physical capture attack is the masquerading attack, which acts as a proper drone. The captured drone returns to the flight formation and wants to report false data to the GCS. The malicious drone will try to run a re-participation protocol to join the drone network at first, but there are two reasons it is hard to pass the conditions in the re-participation protocol. The one reason is that there is no way to modify the encrypted flight data since the attacker does not have the key. The other is that route design considering cross-validation analysis by the neighbors of the captured drone can easily defend this attack, even though the session key is exposed.

It seems to be a threat to the drone environment when the drone near the GCS is physically captured. Following our protocol, the drone near the GCS has low hash chains, so it can generate the child node’s session key with ease. Therefore, the attacker which captures the drone near the GCS seems to disrupt the service by maliciously controlling many child nodes. However, this attack cannot succeed in two possible cases. In the first case, when the drone deployment and the GCS is physically near, the operator can observe the drone network with the naked eye. In this case, the operator will easily notice the physical capture attack and run an emergency mode to return the other nodes. In the second case, even the distance between the drone network and the GCS is too large to see with the naked eye, our system runs the key renewal protocol after the beaconing period. There is not enough time for the captured drone to come back in the beaconing period. Moreover, the returned drone’s re-joining into the deployment will be failed by the node withdrawal and re-participation protocols.

### 5.2. Forward and Backward Secrecy

To satisfy the forward secrecy [15], a drone cannot acquire the group key in any way while it is away from the flight formation. By the key renewal protocol, there is no relationship between the current master session key $k_{i}^{0}$ and the next master session key $k_{i+1}^{0}$. A specific node’s session key is calculated with a hash chain function. The idea using these facts is that the breakaway node cannot generate an expected next session key with its secret information. The backward secrecy is satisfied if the new member joining the group cannot decrypt the ciphertext before then. The proposed system guarantees the backward secrecy due to the similar reason of forward secrecy.

### 5.3. Key Independence

The characteristic of hash function and hash chain function and the key between different session and different hops guarantee independence. Especially since the random nonce is included in the input of the next session key generation, key independence is satisfied. A possible concern is that the node can acquire its children’s session key only using the hash function. However, by considering the concept of a group key, where all group members share the keys, our system satisfies key independence.

### 5.4. Usability Analysis

Guaranteeing the security requirement is indispensable, but satisfying with the concept of usability is also important. As in Table 4, each group key management scheme is designed for its own target system, such as the body area network and wireless sensor network. The attributes and the requirement conditions for the drone network environment are different from other network environments. The systematic attributes for the drone network environment are mobility, multi-hop-based communication, message broadcasting availability, and delegation support. Other algorithms can guarantee some attributes, but our algorithm is the most suitable for the drone environment.

### 5.5. Networking Issue

Drones networks with high mobility in real environments are vulnerable to hidden node problems. This paper basically focuses on the physical capture attack and the key management, but we briefly explain how the proposed system can cope with this problem. In our service scenario, the drone network is configured with a tree topology, and the GCS is a root node. When the key renewal protocol starts, all drones in the network change their antennas to listening mode. Only drones which receive the key renewal message from the GCS (or their parent nodes) can change their antennae into transmission mode. According to this procedure, our protocol follows a top–down communication method. Since this method transmits data from the root node to leaf node following a fixed order, it is possible to say that the method is scheduled. Therefore, our protocol can reduce the hidden node problem without any modification. Additionally, by adding transmission order data into the key renewal message, the hidden node problem can be nearly resolved.

## 6. Evaluation and Simulation Study

In this section, we simply analyze the complexity of the proposed system and some previous work to show that it can satisfy saveless property and low computational overhead and data sending. After then, we show the results of simulation study based on combination of the experimental result and numerical calculation.

### 6.1. Evaluation

Table 5 shows the analysis, where *N* is the total number of drones in the network, *L* is the length of an encrypted group key, *w* is a word size, and *n*, *t* and μ are system parameters and smaller than *N*. We assume that the network topology is binary-tree and that each parent node has two child nodes except the leaf nodes. All criteria in the table are evaluated at the key renewal protocol. Compared to other schemes, the proposed system has the lowest data sending traffic, computational overhead, and storage usage. Following these evaluations, we can assert that our system satisfies saveless property and is suitable for a multi-drone system.

#### 6.1.1. Communication Overhead

Our protocol just broadcast the new key renewal message which contains one integer value, *L* bits encrypted group key, and new topology map with 3Nw bits. In our protocol, the node broadcasts the renewal message with w+L+3Nw only once. In the case of [19], the controller unicasts 2L bits share to *N* nodes. Each node in [20] sends out *n* messages with 2L bits. Following [24], a group manager firstly broadcasts 2logN−1’s new public keys whose size is basically 8L. After that, the group manager broadcasts the *L* bits encrypted message to all nodes. The total communication overhead of [24] is L+8wL(2logN−1).

#### 6.1.2. Computation Overhead

The computation overhead of each node in our protocol is the received message decryption, 2logN times hash chaining, and a message encryption. Following [19], each node receives the encrypted message which contains an update order and a polynomial share. Then it computes the polynomial with $t^{2}$ powers and *t* multiplications. It also computes the group key and share key by running each hash function. In the case of [20], each node updates all pairwise keys from the connected nodes. Therefore, the node must decrypt *N* messages and calculates the key with $o(\mu^{3})$ multiplications and divisions. In [24], each node calculates the new public auxiliary keys for its all parents, and it can get the new group key by decrypting the final public auxiliary key using its secret key.

#### 6.1.3. Storage Overhead

Our system only saves the current session key, *L* bits. However, other algorithms save all sessions’ keys, or lots of information to generate new keys. Especially all members in [24] have a private auxiliary key and logN parents’ public auxiliary key.

### 6.2. Experimentation and Simulation Study

In this subsection, we conduct a simulation study on our key renewal protocol in two perspectives: Computation overhead and energy consumption overhead. Before the simulation, we first implemented our key renewal protocol on Samsung Galaxy A5 2017 with Android 8.0. This system has ARM Cortex-A53 MP8 1.9 GHz CPU and 3 GB LPDDR4 SDRAM. We used this system as a drone control system because of its similar performance to the Odroid XU4 generally used for the drone control system. The Wi-Fi module used in this system is compliant with IEEE 802.11g. To reflect the realistic drone environment, we set a beaconing period as one second.

#### 6.2.1. Computation and Networking Overhead

Following this overhead simulation, we assumed that every parent node has three child nodes. We calculated the total time to complete our key renewal protocol via the hop length of the drone network. After the network topology change, the form of position structure is not changed even when hop count of each node has been changed. Before the simulation, we actually implemented each function and measured their performance on the drone control system. Table 6 shows the experimental result. Following the table, networking delay is larger than computation delay, and it is about 24.5 times larger than the hash chain delay. However, the networking overhead is very small compared to the beaconing period. The maximum possible height of the proposed system is 37 when the beacon period is 1 second. From the experimental result, each function is suitable for a real environment because the system does not actually maintain this height in terms of stability.

**Figure 5:**
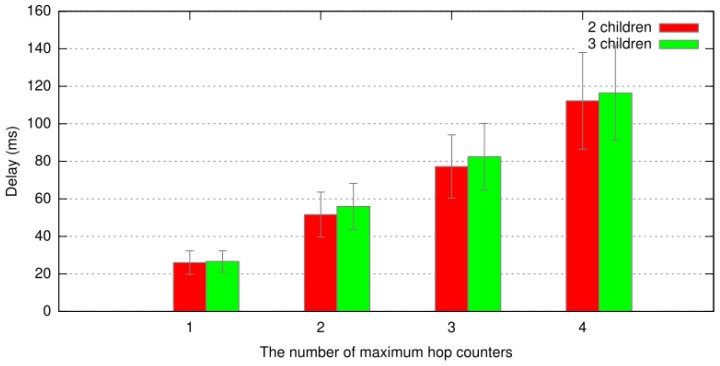
The simulation result based on the experimental data about the key renewal protocol.

Figure 5 shows the result of simulation of the key renewal protocol using the above experimental result. In this figure, as the number of maximum increased, the total delay of the key renewal protocol also increased linearly. However, there was no relationship between the number of child nodes and the total delay. The reason behind these results is that the drone runs one decrypting, several hash functions, and one encrypting function, then broadcasts one encrypted message in our key renewal protocol. As the number of hops increases, the number of hash function execution will also be increasing. However, the delay of hash function is negligible compared to encryption or transmission delay.

#### 6.2.2. Energy Consumption Overhead

Energy analysis is important [25] in our system because the power consumption is a significant factor in the battery-based drone operations. We first measured the actual amount of energy consumed by each function within the key renewal protocol from the drone control device. The result of measured data is shown in Table 7, where KRP means the key renewal protocol. The first column shows the energy consumption of each function with one time activation per one second for 30 minutes. The second column is the ratio comparison, where M600 Pro, which has a 4500 mAh battery and 30 minutes flight time, is the DJI’s drone for industrial purpose. In this result, we can see that our key renewal protocol’s effects on energy consumption are very low compared to flight energy consumption.

**Figure 6:**
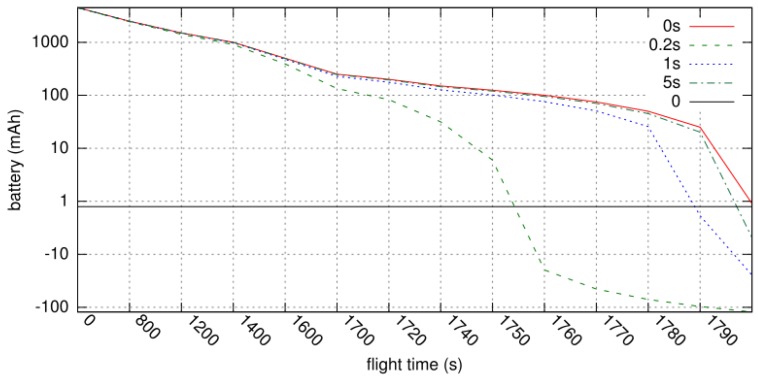
The result for battery power level over time on an operational scenario.

Using the measured energy consumption data, we performed a simulation to analyze the correlation between the actual drones’ flight duration and the beaconing period through the timeline graph. For the simulation, some assumptions were acceptable for actual drone operational scenario: (1) The root node is a mothership drone (a mothership drone is defined as the drone that is directly connected to GCS and can be a delegate of GCS when several drones work at a distance from GCS) which can directly communicate with the GCS; (2) the height of the tree topology is up to 5; (3) all drones except the mothership drone are free to move; (4) there is no drone to withdraw from the formation or re-join. Figure 6 shows the result for the simulation. Compared to the case where the beaconing period is 200 ms, the cases where the beaconing period is 1 second, 5 seconds, and 0 second (no beaconing) can fly 2.17%, 2.62%, and 2.68% longer, respectively. Since each function required for the key renewal protocol basically consumes a small amount of power, it does not affect the actual flight time relatively. Consequently, the proposed algorithms can work well in real-world operating scenarios.

## 7. Conclusions

In this paper, we proposed an authentication and delegation system which is appropriate for a GCS-based multi-drone control system that is necessary for the IoT environment to compose and operate it. Compared to a general group key management scheme, the proposed system is focused on the physical capture attack that is a critical threat to the multi-drone system. Therefore, the proposed system is based on the idea that storing secret data, such as a group key, flight data, and operational intelligence, will be a minimized, so-called saveless property. The proposed key distribution protocol is based on the saveless property. It also includes key renewal and a node re-participation protocol to apply unconstrained mobility, which is the core attribute of drone environment. We additionally proposed the drone delegation system for delegating control right to other GCSs by the drone’s operation and position. The complexity analysis shows that the algorithm can reduce storage space without any additional overhead. The simulations based on the data from experiments verify that our system is suitable in the real-world environment.

As future work, we would like to reduce the proposed key management and delegation protocol to work with very small IoT devices that have limited computation power, and to find a proper key renewal period that satisfies a low computation cost and more security guarantee.

## Figures and Tables

**Table 1:** Notations for the communication protocol and key renewal protocol.

$E_{s}\left(msg,k\right)$	Encrypt plaintext msg using symmetric key *k*
$D_{s}\left(emsg,k\right)$	Decrypt ciphertext emsg using symmetric key *k*
$h^{n}\left(\cdot \right)$	Hash chains with *n* rounds
$id_{x}$	Identification of drone *x*
$pid_{x}$	Identification of drone *x*’s parent
$h_{i,id}$	A hop count of drone id at session *i*
$r_{x}$	A random nonce generated by *x*
S	A set of sending nodes
R	A set of next-hop receiving nodes
$nl_{x}$	A list of neighbor of node *x*

**Table 2:** Notations for withdrawal and re-participation.

$N_{b}$/$N_{d}$	The breakaway node/the first discovered node
$fd_{x}$	A drone flight data with temporary session *x*
$t_{x}$	A timestamp
$rpos_{x,t}$	The real position of node *x* at time *t*
$spos_{x,t}$	The scheduled position of node *x* at time *t*
$d_{th}$	The distance threshold which follows the system settings
$t_{th}$	The breakaway time threshold
$\tilde{k}$	One-time session key between $N_{b}$ and GCS
λ	Maximum of temporary session number of $N_{b}$
$h^{n}\left(x,y\right)$	Hash chain functions each hashing input is previousresult *x* concatenated *y* for *n* times
$e_{th}$	An average cross track error threshold
$\Delta_{th}$	Distance threshold during one temporal session

**Table 3:** Notations used in the delegation system.

$\mathrm{Sig}\left(x,y\right)$	Signing a message *x* with key *y*
$sk_{x}$/$pk_{x}$	The private key of *x*/the public key of *x*
$f\left(x,y\right)$	Keyed hash function (*x* is message, *y* is key)
$k_{x-y}$	Shared key between node *x* and *y*
$c_{\max}$	A maximum command counter
A	A set of nearest nodes for each delegated node

**Table 4:** Comparison between our proposed system and some previous works.

Criteria	Ours	GDP [19]	C-PCGR [20]	Chen’s [24]
Symmetric key-based	√	√	√	×
Capture resistant	√	×	√	×
For-/backward secrecy	√	Δ	×	√
Key independence	√	×	√	×
Multi-hop communication	√	×	√	√
Msg. broadcasting support	√	√	×	√
Mobility support	√	Δ	×	Δ
Delegation support	√	×	×	×

**Table 5:** Complexity analysis between our proposed system and some previous works.

Criteria for Each Drone	Ours	GDP [19]	C-PCGR [20]	Chen’s [24]
Data sent (bits)	w+L+3Nw	2NL	2nL	L+8wL(2logN−1)
computational overhead	1 encryption, 1 decryption, 2logN hashings	1 decryption, 2 hashings, *t* multiplications and $t^{2}$ powers over F(q)	*N* decryptions, $o(\mu^{3})$ multiplications and divisions over F(q)	logN public key encryptions, 1 public key decryption
Storage usage (bits)	*L*	$\left(\frac{N(N-1)}{2}+1\right)L$	(2N+1)(t+1)L	L(1+logN)

**Table 6:** The experiment result for computation and networking overhead.

	Computation		Networking
	hash chain	AES	broadcast
mean (ms)	1.10	3.83	26.9
std. (ms)	0.69	1.02	5.93

**Table 7:** The simulation result for energy consumption.

	Hash	enc/dec	Trans.	KRP
30 min (mAh)	1.23	2.66	15.95	23.73
M600 Pro (%)	0.027	0.059	0.35	0.53
